# Supplementary material for: Structural and Morphogenetic Characteristics in Paspalum notatum: Responses to Nitrogen Fertilization, Season, and Genotype
Source: Plants (Basel). 2023 Jul 13;12(14):2633. doi: 10.3390/plants12142633 (PMC10383767; doi:10.3390/plants12142633)
Supplement: Supplementary file 1 [file plants-12-02633-s001.zip › plants-2392698-supplementary.pdf]

Table S1: Sources of variation, degrees of freedom (df), mean square (MS), and significances (Sign.) for structural and morphological variables: herbage mass (HM), herbage accumulation rate (HAR), tiller density (TD), tiller weight (TW), leaf blade length (LBL), leaf blade width (LBW), number of total leaves (NTL), number of elongating leaves (NEL), number of unfolded leaves (NUL), phyllochron (Phyll.), leaf elongation rate (LER), leaf elongation time (LET), and leaf half-life (LHL).

**Source of variation**	**HM**			**HAR**			**TD**			**TW**			**LBL**			**LBW**		
	df	MS	Sign.	df	MS	Sign.	df	MS	Sign.	df	MS	Sign.	df	MS	Sign.	df	MS	Sign.
**Period**	3	2077484	**	3	525095	**	3	949177	**	3	1410204	**	3	6820	**	3	0,235	**
**Nitrogen**	1	591336	**	1	148182	**	1	5155555	**	1	46445	**	1	529	**	1	0,091	**
**Genotype**	9	15495	ns	9	3827	ns	9	116914	*	9	42294	**	9	375	**	9	0,042	**
**Period*Nitrogen**	3	282171	**	3	70294	**	3	458201	**	3	21719	*	3	876	**	3	0,014	*
**Genotype*Period**	27	30868	**	27	7385	**	27	59875	*	27	11852	*	27	72,1	**	27	0,009	**
**Nitrogen*Genotype**	9	3389	ns	9	837	ns	9	13235,5	ns	9	6738	ns	9	32,3	ns	9	0,006	ns
**Genotype*N*Period**	27	14042	ns	27	3468	ns	27	46009,2	ns	27	7400	ns	27	21,4	ns	27	0,004	ns

ns, not significant at 0.05 probability level
* Significant at the 0.05 probability level.
** Significant at the 0.01 probability level.

Table S1: Continue

**Source of variation**	**NTL**			**NEL**			**NUL**			**LER**			**LET**			**Phyll.**			**LHL**		
	df	MS	Sign.	df	MS	Sign.	df	MS	Sign.	df	MS	Sign.	df	MS	Sign.	df	MS	Sign.	df	MS	Sign.
**Period**	2	27,035	**	2	2,500	**	2	8,972	**	1	0,070	*	1	162223	**	2	51263	**	2	1210208	**
**Nitrogen**	1	0,060	ns	1	0,018	ns	1	0,120	ns	1	1,513	**	1	42720	**	1	17060	**	1	27018	ns
**Genotype**	9	6,274	*	9	0,442	*	9	0,297	*	9	0,065	*	9	16636	**	9	9834	*	9	40802	**
**Period*Nitrogen**	2	1,087	ns	2	0,084	ns	2	0,285	*	1	0,010	ns	1	169	ns	2	1019	ns	2	71680	**
**Genotype*Period**	18	1,476	*	18	0,125	*	18	0,056	ns	9	0,011	ns	9	1920	ns	18	1384	*	18	12130	ns
**Nitrogen*Genotype**	9	0,980	ns	9	0,073	ns	9	0,065	ns	9	0,017	ns	9	3933	*	9	844	ns	9	13312	ns
**Genotype*N*Period**	18	0,463	ns	18	0,040	ns	18	0,068	ns	9	0,011	ns	9	1675	ns	18	449	ns	18	6617	ns

ns, not significant at 0.05 probability level
* Significant at the 0.05 probability level.
** Significant at the 0.01 probability level.
